# Hospital Expenditure and Efficiency

**Published:** 1905-03-25

**Authors:** 


### Editor's Letter-Box.

[Our Correspondents are reminded that prolixity is a great bar to publication, and that brevity of style and conciseness of statement greatly facilitate early insertion.]

### HOSPITAL EXPENDITURE AND EFFICIENCY.

Mr. Sydney Holland has written a long letter on this subject which came to hand too late to be dealt with this week.

